# Raman Spectroscopy and Imaging Reveal the Effect of β‑Carotene Supplementation on Brain Cancer Cells

**DOI:** 10.1021/acs.biochem.5c00231

**Published:** 2025-08-20

**Authors:** Karolina Jarczewska, Monika Kopeć, Halina Abramczyk, Jakub Maciej Surmacki

**Affiliations:** Faculty of Chemistry, Institute of Applied Radiation Chemistry, Laboratory of Laser Molecular Spectroscopy, Lodz University of Technology, Wroblewskiego 15, Lodz 93-590, Poland

## Abstract

Dietary β-carotene is the most common carotenoid in the world. Naturally occurs in vegetables and fruits (e.g., carrots, tomatoes). Recently, β-carotene has been studied for its effects on the human body; however, the effect of this carotenoid on brain tumor metabolism at the cellular level is still unknown. Here, we consider whether β-carotene influences brain tumor cell metabolism and, if so, whether this effect stimulates or inhibits tumor growth. To find out the effect of β-carotene on brain cells (normal human astrocytes, astrocytoma, and glioblastoma), we applied Raman spectroscopy and imaging. We focused our analysis on biological changes in particular cell organelles such as the nucleus, mitochondria, lipid droplets/endoplasmic reticulum, and cytoplasm. Our Raman results demonstrated that cancer cell metabolism is altered following β-carotene supplementation, as reflected in changes to Raman bands associated with cytochrome *c* (1310 and 1583 cm^–^^1^), lipids (1337 and 1444 cm^–^^1^), and proteins (1337 and 1654 cm^–^^1^). The response to supplementation is different not only for normal cells compared to cancer cells (the effects vary depending on the cell type) but also for supplementation timing and doses.

## Graphical abstract

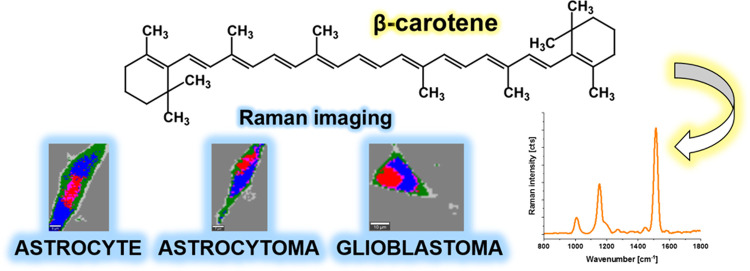

## Introduction

Carotenoids are fat-soluble natural pigments synthesized by plants and photosynthetic and nonphotosynthetic organisms such as bacteria, yeasts, and molds.^1^ It has been shown that animals cannot synthesize carotenoids de novo, which indicates that diet rich in carotenoids is crucial for the maintenance of health.^1^ Carotenoid chemical structure represents a polyene carbon chain core, ending with cyclic or acyclic groups. They can be divided into two main groups: carotenes and xanthophylls, which differ in the presence of at least one oxygen atom in xanthophylls.^2,3^

The most popular carotenoid in diets worldwide is β-carotene, which naturally occurs in vegetables and fruits (e.g., carrots, tomatoes, cantaloupe, red bell peppers, and pumpkins).^4,5^ In plants, β-carotene accumulates in plastids.^6^ There are plenty of β-carotene industrial applications from being a pigment in the food industry, to acting as a drug in the cosmetics industry.^7–9^ The enormous popularity of β-carotene is due to various options for obtaining it from natural sources, chemical synthesis, or synthesis by microbial.^10^ In animals, β-carotene is a precursor of vitamin A converted from provitamin A.^11^ The major property of β-carotene, among other antioxidants, is to protect the skin from the impact of UV radiation and improve eyesight. Moreover, it improves the production of collagen in mucous membranes, increases bone mass, and stimulates the immune system.^1^ Many publications indicate that β-carotene is a valuable factor in cancer prevention.^12–14^ Due to its nonpolar structure, β-carotene does not dissolve in water but dissolves easily in lipids, thereby penetrating through lipid membranes.^14^ It has been proven by Abramczyk et al. and Surmacki et al. recent papers that compounds from the retinoid group, directly related to carotenoids, are crucial in modulating processes at the cellular level, for example by influencing the expression of cytochrome *c*.^15–17^ Previous results of Brozek-Pluska et al. showed that β-carotene is an important protective factor against oxidative stress on human normal colon cells.^18^ Moreover, β-carotene has been found to prevent type 2 diabetes, obesity, photosensitivity disorders, and cardiovascular diseases.^19,20^ A recent study shows that β-carotene as a carotenoid inside the human body acts by reacting with radicals, cell growth regulation, or modulating gene expression.^21–23^ Furthermore, β-carotene has shown an effect on modulating microRNA (miRNA) expression.^24^

Metabolism of β-carotene occurs in human liver and intestines, where β-carotene is symmetrically cleaved by β-carotene-15,15′-oxygenase at the 15,15′ bond, which generates two molecules of retinaldehyde. Retinaldehyde can be oxidized to retinoic acid, which then can be oxidized by the cytochromes P450 26 family to 4-oxo-retinoic acid. Alternatively, retinaldehyde might be converted into retinol by alcohol dehydrogenases or retinol dehydrogenases and then can be further converted into retinyl esters. Another mechanism is to cleave β-carotene by the β-carotene-9,10′-oxygenase to 2 apo-carotenals. In summary, carotenoid metabolism generates retinoids.^6,25,26^ The β-carotene has two isomeric forms: cis and trans isomers, which have different metabolisms, but due to a significant quantitative excess only the *trans*-β-carotene is considered.^25^

Brain cancer is one of the most lethal types of cancer due to challenges with the crossing of drugs across the blood–brain barrier and the surgical removal of brain tissue around the tumor because the structure of the brain is more complex than that of other organs, and every part of it plays an important role in the functioning of the body. When a cell becomes cancerous, the whole metabolism changes.^27^ There are more than 120 different types of brain tumors, but even benign tumors may be dangerous due to their localization or their size (easy growth). Every tumor in the intratemporal space creates pressure on the healthy parts of the brain and/or spreads to those areas. The origin of brain tumors is often from the metastases from other organs like breast, colon, kidney, lung, or skin.^28,29^ Brain tumors can arise in any part of the brain and are often named after their location, such as meningiomas, which arise in the meninges, pituitary tumors from the pituitary gland, medulloblastomas of the cerebellum or brainstem, and skull base tumors, which develop beneath the brain in the base of the skull.^30^ Our analysis focuses on astrocytoma (CRL-1718) and glioblastoma (U-87 MG) cells. Statistics show that brain tumors are more common in children and adolescents than in adults.^31,32^ Attempts to qualitatively analyze a brain tumor to determine the degree of aggressiveness most commonly include a biopsy of the sampled tumor specimen.^30^ However, a standard biopsy is a time-consuming process (patients wait approximately a few weeks for the result), so finding a fast technical analysis is still required. Here, we propose label-free Raman spectroscopy (RS) as a fast, quick technique that gives biochemical information. RS is a technique that provides complex and detailed information about chemical structure and quantitative and qualitative composition of analyzed samples based on analysis of scattered light. The principle of this technique is the light interaction with the sample.^33,34^ A result of those interactions is light scattering, observed in a Raman spectrum. The Raman spectrum is a molecular fingerprint of the analyzed sample. There are plenty of applications for RS and imaging: semiconductors analysis, catalysts, glasses, gels, clays, environmental materials, archeological materials, polymers, and biological materials (proteins, nucleic acids, lipids, carotenoids).^34–37^

β-carotene, a dietary precursor of vitamin A, is recognized for its antioxidant properties and its potential role in modulating redox homeostasis. Its capacity to scavenge reactive oxygen species (ROS) is particularly relevant, as oxidative stress contributes to DNA damage and tumor initiation. In addition, β-carotene may influence key signaling pathways involved in proliferation, apoptosis, and immune regulation, processes commonly dysregulated in brain tumors. Although current evidence remains inconclusive, investigating this relationship may help uncover new preventive or therapeutic strategies. Although the effects of carotenoids on the human body are seemingly well understood, there are still gaps in our knowledge regarding the mechanisms by which β-carotene acts on individual human brain cells under healthy conditions and during cancer progression with varying levels of aggressiveness. Therefore, we proposed in this work the methodology of applying RS and imaging to elucidate the effects of β-carotene on brain cells and to know how it varies for different degrees of tumor aggressiveness, β-carotene concentration, and incubation time.

The cell lines used in our study—normal human astrocytes (NHA), astrocytoma (CRL-1718), and glioblastoma (U-87 MG)—are well-established and commercially available models representing a gradient of tumor progression and aggressiveness, from nontumorigenic astrocytes, through lower-grade astrocytoma, to highly malignant glioblastoma. These lines exhibit defined molecular and phenotypic characteristics and are widely accepted in preclinical neuro-oncology research as reliable systems for studying cancer biology.^38–40^ In particular, U-87 MG displays high proliferative and invasive potential with extensive cytogenetic alterations, whereas CRL-1718 is considered less aggressive, providing a useful contrast to the nontumorigenic NHA. The distinct biological behaviors and metabolic profiles of these cell types have also been characterized using Raman-based methods,^41,42^ supporting their suitability for mechanistic studies. Employing this panel enables the controlled investigation of tumor-related processes, including metabolic responses to compounds such as β-carotene, across different malignancy levels before in vivo or clinical validation.

## Materials and Methods

### Chemicals

The β-carotene (no. 1065480, United States Pharmacopeia (USP) Reference Standard) was purchased from Merck Life Science, Darmstadt, Germany.

### Cell Culture and Preparation for Microscopy

Normal human astrocytes (NHA), astrocytoma (CRL-1718), and glioblastoma (U-87 MG) were purchased from Lonza and ATCC. NHA during the first three passages was cultured with Astrocytes Growth Medium (ABM no. CC-3187 and Kit no. CC-3186), and then, the medium was exchanged to Dulbecco’s modified Eagle medium (DMEM, Merck Life Science, Darmstadt, Germany no. D0819). In our preliminary experiments, we observed limited cell growth when culturing NHA cells in the manufacturer-recommended medium (AMB). To improve cell viability and proliferation, we evaluated an alternative medium, DMEM, which has been successfully applied to similar cell lines. This substitution resulted in markedly improved cell growth and morphology, enabling the collection of a sufficient number of viable cells for the planned experiments. Therefore, we adopted the modified culture conditions to ensure the experimental reliability and reproducibility. The CRL-1718 cell line was cultured with RPMI 1640 (Merck Life Science, Darmstadt, Germany, no. R8758). The U-87 MG cell line was cultured with Minimum Essential Medium Eagle Medium EMEM (Merck Life Science, Darmstadt, Germany, no. M2279). For each cell line, the medium was changed every 2 to 3 days and the passage was done once or twice per week. Each medium was prepared by adding Fetal Bovine Serum (FBS no. 30-2020 ATCC) to a final concentration of 10%. Cells were cultured in T75 flasks (TPP - Techno Plastic Products, Trasadingen, Switzerland no. 90076) and in a humidified atmosphere at 37 °C with 5% of CO_2_. Twenty-four hours before the supplementation with β-carotene, cells were seeded into the CaF_2_ substrate (Crystran Ltd., Poole, UK; CaF_2_ Raman grade optically polished window 25 mm diameter × 1 mm thick, no. CAFP25-1R, Poole, UK) in a 35 mm Petri dish. A 10 mM stock β-carotene solution was prepared by dissolving β-carotene in dimethyl sulfoxide (DMSO, Merck Life Science, Darmstadt, Germany no. D84180). β-carotene is a hydrophobic (lipophilic) molecule and is poorly soluble in an aqueous environment.^25^ To overcome this challenge, we have proposed a method for the preparation of β-carotene solution by shaking β-carotene with Fetal Bovine Serum (FBS).^43,44^ In FBS, β-carotene may bind to serum proteins, such as albumin or lipoproteins (HDL and LDL), which act as carriers for hydrophobic molecules. This interaction can improve the apparent solubility of β-carotene in an aqueous medium. β-Carotene was first dissolved in DMSO to prepare a 10 mM stock solution. A 300 μL amount of this stock was then mixed with 300 μL of fetal bovine serum (FBS) by shaking to enhance solubility. The resulting 5 mM β-carotene/FBS solution was diluted into cell line-specific culture media to achieve final concentrations of 1 μM (2 μL stock per 10 mL medium), 10 μM (20 μL stock per 10 mL medium), and 50 μM (100 μL stock per 10 mL medium). After 24 h of growth, cells were supplemented with a β-carotene solution in cell line-dedicated media in the following concentrations: 1, 10, and 50 μM. Cells were incubated with β-carotene for 24 and 48 h before the measurement. The concentrations of 1, 10, and 50 μM and incubation times of 24 and 48 h were selected based on previously published studies investigating the effects of β-carotene in various cell models, including human cancer lines.^45,46^ These concentrations are within the biologically relevant range and reflect physiologically achievable doses under the experimental conditions.

After the incubation, cells were fixed with 4% formalin solution (neutrally buffered) and measured in phosphate-buffered saline (PBS, no. 10010023, Gibco, Waltham, MA, USA). Each cell line was analyzed as follows: control, 1 μM at 24 and 48 h, 10 μM at 24 and 48 h, and 50 μM at 24 and 48 h.

### Raman Spectroscopy and Imaging

Raman spectra and images were obtained using an Alpha 300 RSA+ confocal microscope (WITec, Ulm, Germany). Raman data were collected in two spectral ranges: 2700–3100 cm^–1^ with 0.3 s integration time and 400–1800 cm^–1^ with 0.5 s integration time using EMCCD, 532 nm laser wavelength (10 mW), and 40× water immersion objective (NA = 1.0). These two spectral ranges were selected to offer a thorough understanding of the changes occurring within the cell. Raman imaging of a single cell (typical dimensions approximately 34 × 40 μm with 1 μm spatial resolution, 1400 Raman spectra) required approximately 12 min for the high Raman shift range (2700–3100 cm^–^^1^) and 21 min for the low range (400–1800 cm^–^^1^). Consequently, total imaging time per cell was approximately 35 min. Detailed information about the Alpha 300 RSA+ confocal microscope is available in the given reference.^16,47^ The number of Raman spectra used for averaging in control samples was 3605 for NHA, 2395 for CRL-1718, and 2150 for U-87 MG cells. For β-carotene-treated NHA cells, the number of spectra was: 1 μM - 2195 (24 h), 2466 (48 h); 10 μM - 2708 (24 h), 2128 (48 h); 50 μM - 2394 (24 h), 2002 (48 h). For CRL-1718 cells: 1 μM, 2313 (24 h), 2543 (48 h); 10 μM, 2191 (24 h), 1867 (48 h); 50 μM, 1804 (24 h), 2648 (48 h). For U-87 MG cells: 1 μM - 2086 (24 h), 1904 (48 h); 10 μM - 1158 (24 h), 1669 (48 h); 50 μM - 1570 (24 h), 897 (48 h). A total of approximately 44693 Raman spectra, acquired from 84 individual cells across all experimental conditions, were included in the subsequent analysis.

As part of the data preprocessing, cosmic rays’ removal (filter size 2, dynamic factor 10), smoothing (Savitzky-Golay, order 4, derivative 0), and the removal of background were performed. To distinguish individual cell organelles, a cluster analysis with seven clusters was done in WITec Project Plus Four 4.1. Briefly, the cluster analysis is an unsupervised exploratory approach used to group observations based on intrinsic similarities, in this study, Raman spectral features. The method aims to maximize homogeneity within each cluster while preserving the distinctiveness between clusters. The partitioning of n observations (*x*) into *k* clusters (*S*, where *k* ≤ *n*) is performed to minimize intracluster variance (Var), according to the following principle:

$$\arg\min_{S}\sum_{i=1}^{k}\sum_{x\in S_{i}}∥x\mu_{i}{∥}^{2}=\arg\min_{S}\sum_{i=1}^{k}|S_{i}|\mathrm{Var}S_{i}$$

where μ_*i*_ is the mean of points *S_i_*. Organelles were identified based on spectral similarity patterns revealed through cluster analysis, and these assignments were independently validated using microscopic imaging of the corresponding cells, as previously described in refs 16 and 47–49 . The cluster analysis was performed separately for each individual cell rather than collectively across all cells. The resulting clusters correspond to specific cellular compartments, including the nucleus (red), mitochondria (magenta), lipid droplets and endoplasmic reticulum (blue), oleic lipid droplets (orange), cytoplasm (green), cell membrane (light gray), and the extracellular region (dark gray). ANOVA, a one-way statistical analysis, was done using the Origin Pro 2021 program. The significant difference (*p*-value ≤ 0.05) was presented as an asterisk [*].

The partial least-squares discriminant analysis (PLS-DA) was performed on vector-normalized and mean-centered low range Raman spectral data (400–1800 cm^–1^) using the PLS_Toolbox in MATLAB (MathWorks, Natick, MA, USA) (Figure S4). The Pearson correlation analysis was conducted in OriginPro 2021 (OriginLab Corporation, Northampton, MA, USA) on the normalized data set (Table S1). A confidence level of 95% (*p* < 0.05) was applied to all statistical tests presented in the Supporting Information.

### UV–Vis Spectra of β-Carotene

UV–vis spectra of β-carotene were collected in a range of 200–800 nm on a Lambda 750 PerkinElmer (Waltham, MA, USA) spectrometer. β-Carotene/DMSO solutions were measured in a 1 mm quartz cuvette.

## Results

In this paper, we applied RS and Raman imaging to determine the effects of β-carotene supplementation on brain cells. Figure 1 shows the structure of β-carotene and Raman and UV–vis spectra of β-carotene solution in DMSO.

**Figure 1:**
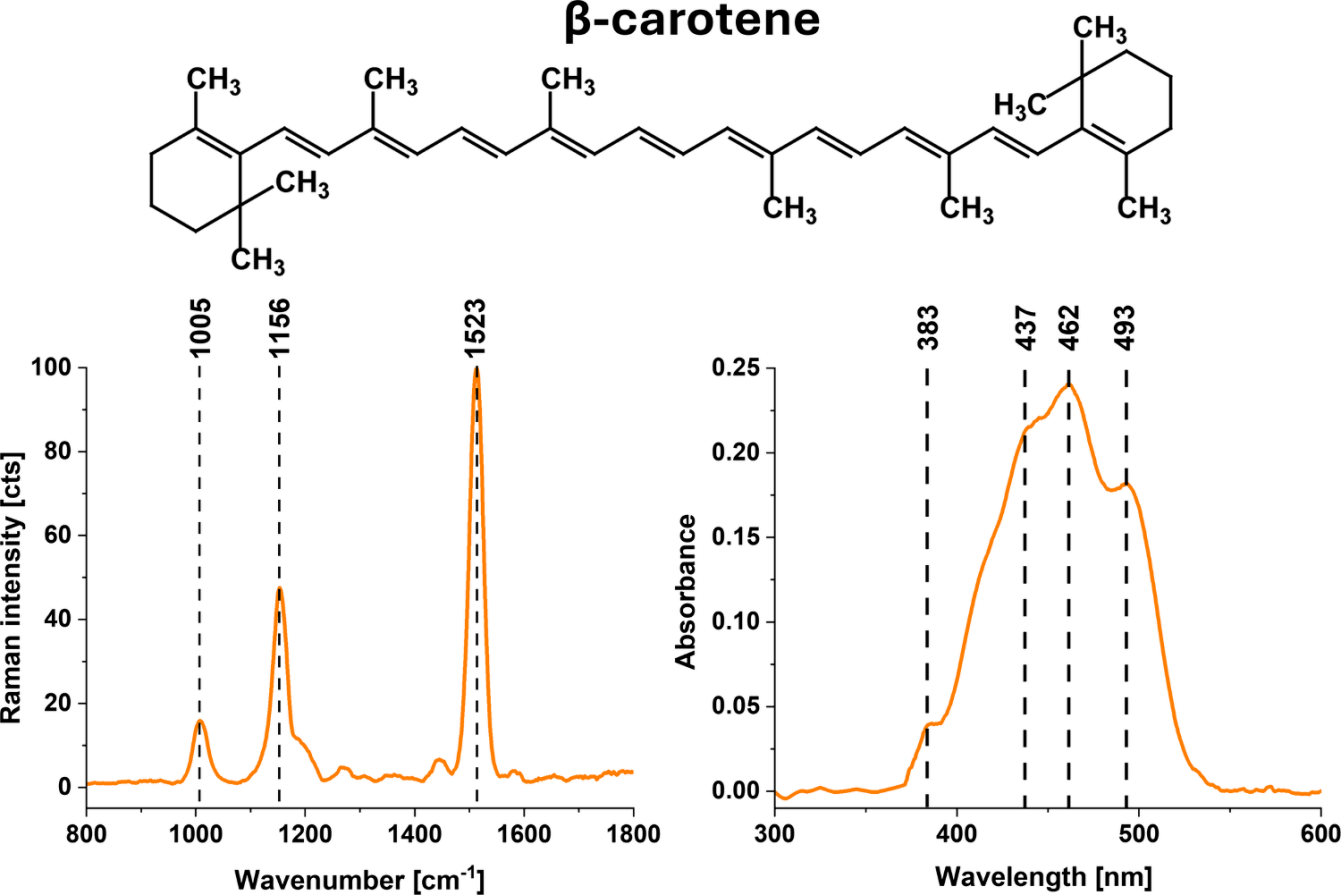
Molecular structure and Raman and UV–vis spectra of β-carotene. Raman spectrum: 10 mM β-carotene in DMSO; UV–vis spectrum: 10 mM β-carotene in DMSO.

We have focused on the analysis of normal human astrocytes (NHA), astrocytoma (CRL-1718), a mildly aggressive brain tumor, and glioblastoma (U-87 MG), a highly aggressive brain tumor without and supplemented with β-carotene.

Figure 2 presents typical Raman imaging of a single unsupplemented NHA, CRL-1718, and U-87 MG cells, Raman images of particular cell organelles as a nucleus, mitochondria, lipid droplets/endoplasmic reticulum, cytoplasm, and cell membrane, and corresponding Raman spectra.

**Figure 2:**
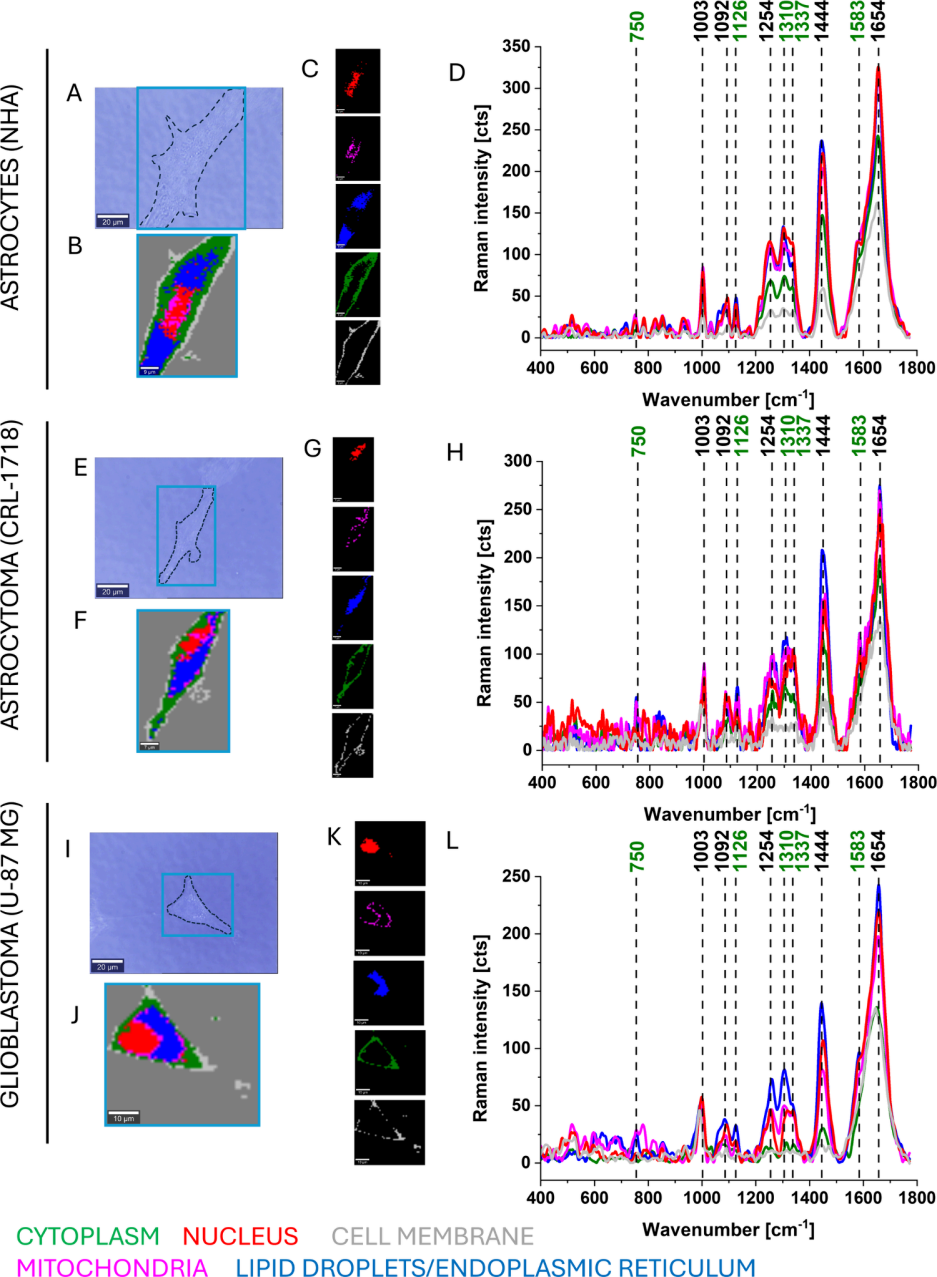
Typical Raman imaging of normal human astrocyte (NHA), astrocytoma (CRL-1718), and glioblastoma (U-87 MG) cells. (A, E, I) Microscope images of the cell; (B, F, J) Raman images of the cell; (C, G, K) Raman images of particular cell organelles; (D, H, L) representative Raman spectra of each cell organelle: nucleus (red), mitochondria (magenta), lipid droplets/endoplasmic reticulum (blue), cytoplasm (green), and cell membrane (light gray). Raman spectra were measured in a range of 400–1800 cm^–1^ with an integration time of 0.5 s at 10 mW and 532 nm. Colors on the spectra correspond to colors on Raman images.

To verify the influence of β-carotene supplementation on human brain cell lines, we have conducted analogous Raman measurements and data analysis. Figure 3 presents representative Raman data obtained from NHA, CRL-1718, and U-87 MG cells supplemented with β-carotene at concentrations of 1, 10, and 50 μM after 24 h. Figure S2 presents the effects of β-carotene supplementation after 48 h.

**Figure 3:**
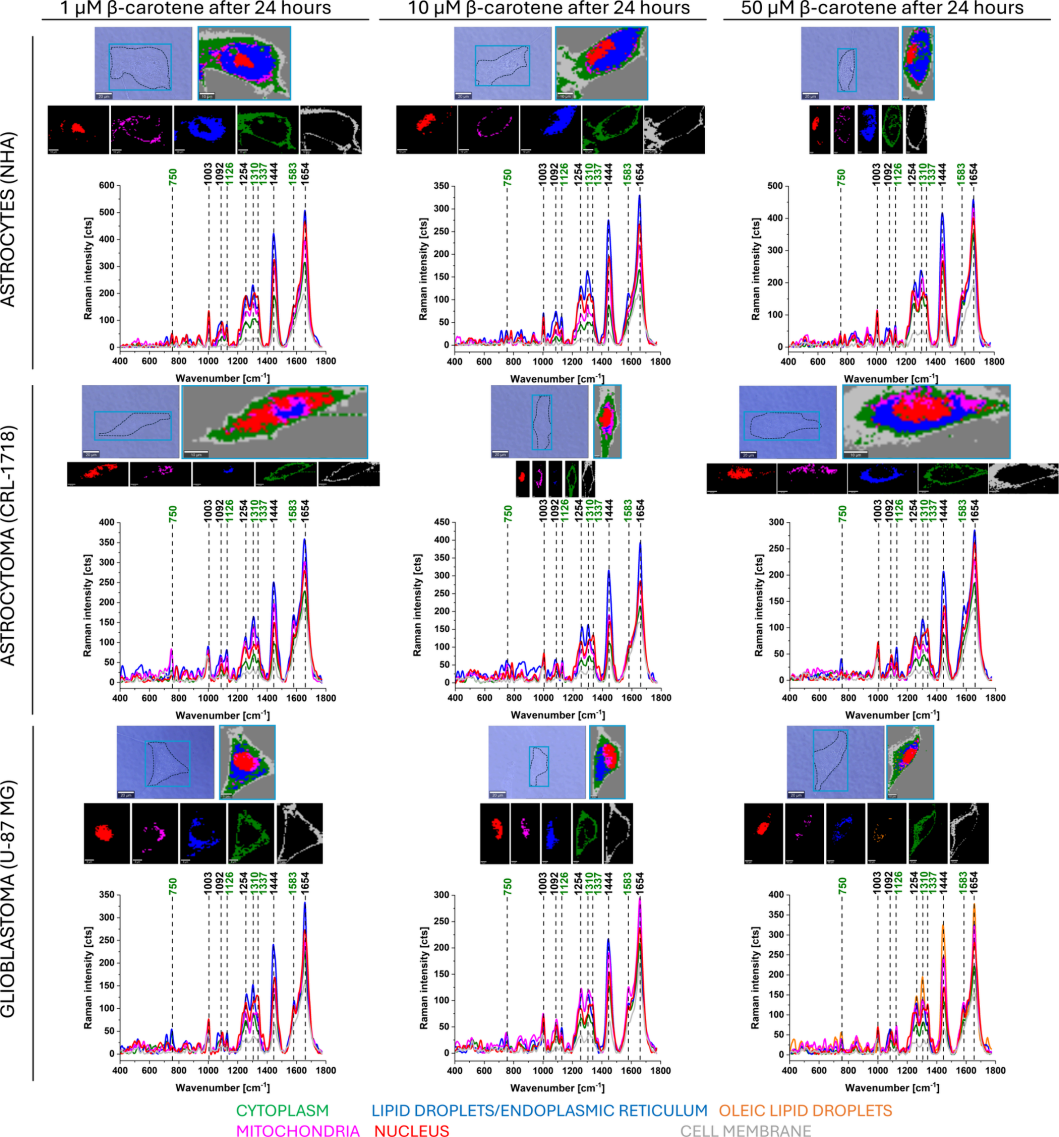
Typical Raman imaging of a normal human astrocyte (NHA), astrocytoma (CRL-1718), and glioblastoma (U-87 MG) cells supplemented with β-carotene at concentrations: 1 μM, 10 μM, 50 μM, and time of incubation 24 h. Microscope images of the cell; Raman images of the cell; Raman images of particular cell organelles; representative Raman spectra of each cell organelle: nucleus (red), mitochondria (magenta), lipid droplets/endoplasmic reticulum (blue), cytoplasm (green), and cell membrane (light gray). Raman spectra were measured in a range of 400–1800 cm^–1^ with an integration time of 0.5 s at 10 mW and 532 nm. Colors on the spectra correspond to colors on Raman images.

Figures 2 and 3 show that Raman techniques give us the ability to analyze the cell organelles at the micrometer (μm) scale. In the fingerprint spectral region, we observe strong Raman bands at 750, 1003, 1092, 1126, 1254, 1310, 1337, 1444, 1583, and 1654 cm^–1^. Raman bands at 750, 1126, 1310, and 1583 cm^–1^ correspond to cytochromes (heme proteins). The band at 1003 cm^–1^ corresponds to the phenylalanine. The band at 1092 cm^–1^ is assigned to DNA. Bands at 1444 and 1254 are assigned to fatty acids/lipids and at 1337 and 1654 cm^–1^ to lipids and proteins.^17,49–59^ Detailed assignments are listed in Table 1.

**Table 1:** Raman Vibrational Mode Assignments for the Analyzed Bands^14,31–41^

Raman band wavenumber [cm^–1^ **]**	Raman vibrational mode assignment
715/716	adenine/CN–(CH_3_)_3_ (lipids)/choline group
750	tryptophan (proteins)/cytochrome *c*
851/855	polysaccharides/tyrosine/proline
907	formalin contamination peak on fixed tissues
994	C–O ribose/C–C vibrations
1003/1005	C–C stretching/phenylalanine (proteins)
1080	phospholipids/C–C stretching
1092	O–P–O backbone stretching in DNA/phospholipids
1126	C–N stretching in proteins/cytochrome *c*
1154/1156	C–C, C–N stretching in proteins/carotenoids
1254	PO_2–_ asymmetric stretching in lipids
1310	guanine-ring breathing mode in DNA/cytochrome *c /m*ixed fatty acid chains
1337	Lipids and proteins
1444	CH_2_ and CH_3_ deformation vibrations in lipids/Mixed amide I protein
1523/1527	(−C=C−) in carotenoids
1583	cytochrome *c*
1654	C=C stretching in lipids and proteins/amide I
2848	symmetric stretching of CH_2_ in lipids, triglycerides, phospholipids
2870	symmetric stretching of CH_3_ of lipids, CH_2_ asymmetric stretch of lipids and proteins
2880	symmetric stretching of CH_2_ and CH_3_, and asymmetric stretching of CH of lipids
2900	stretching of CH_3_ in lipids or proteins; CH stretch
2926	CH stretch of lipids and proteins, CH_3_ stretching vibrations, large aromatic ring system
2930	symmetric and asymmetric stretching of CH_3_ in proteins
2964	asymmetric stretching of CH_3_
2982	asymmetric stretching of methoxy
3010	unsaturated =CH stretch

From Figures 2 and 3, one can see that the bands at 1254, 1310, 1444, and 1654 cm^–1^ have the highest intensity for lipid droplets/endoplasmic reticulum. From Figure 3, it should be noted that after supplementation with 50 μM β-carotene in U-87 MG cells, oleic lipid droplets appeared (orange color). Characteristic Raman bands for oleic acid are 1080, 1264, 1300, 1440, and 1654 cm^–1^,^60^ which are present in analyzed spectra of brain cells (Figures 2 and 3). The main difference between lipid droplets and oleic lipid droplets is the spectral profile of Raman bands in a region of 2800–3000 cm^–1^. In the biological viewpoint, the oleic lipids contain more oleic acids than the typical lipid droplets.

Based on the literature reports on β-carotene metabolism,^6,61^ we decided to focus further analysis on the lipid droplets/endoplasmic reticulum (Figure 4), cell nucleus (Figure 5), mitochondria (Figure 6), and cytoplasm (Figure S1). Figures 4–6 and Figure S1 present the offset of Raman intensity and comparisons between the average Raman spectra derived from all analyzed cells under each experimental condition (control and supplemented cells with 1, 10, and 50 μM of β-carotene after 24 and 48 h of incubation).

**Figure 4:**
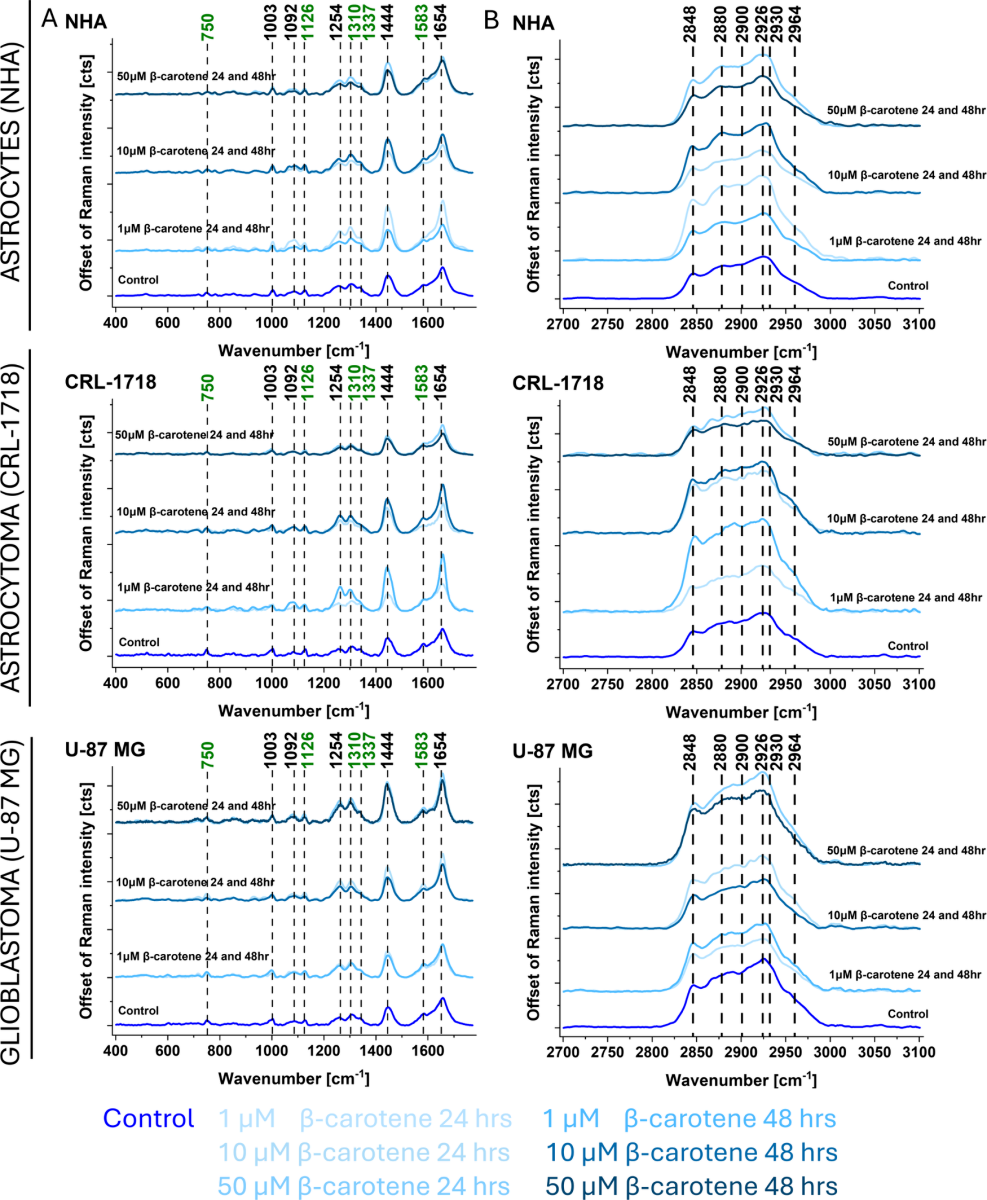
Offset of an average Raman spectrum for cell lipid droplets/endoplasmic reticulum (NHA, CRL-1718 and U-87 MG). Ranges: (A) 400–1800 cm^–1^ and (B) 2700–3100 cm^–1^. Colors on the spectra correspond to control and supplemented cells: 1, 10, and 50 μM of β-carotene after 24 and 48 h. Cytochrome *c* is represented by the Raman bands marked in green.

**Figure 5:**
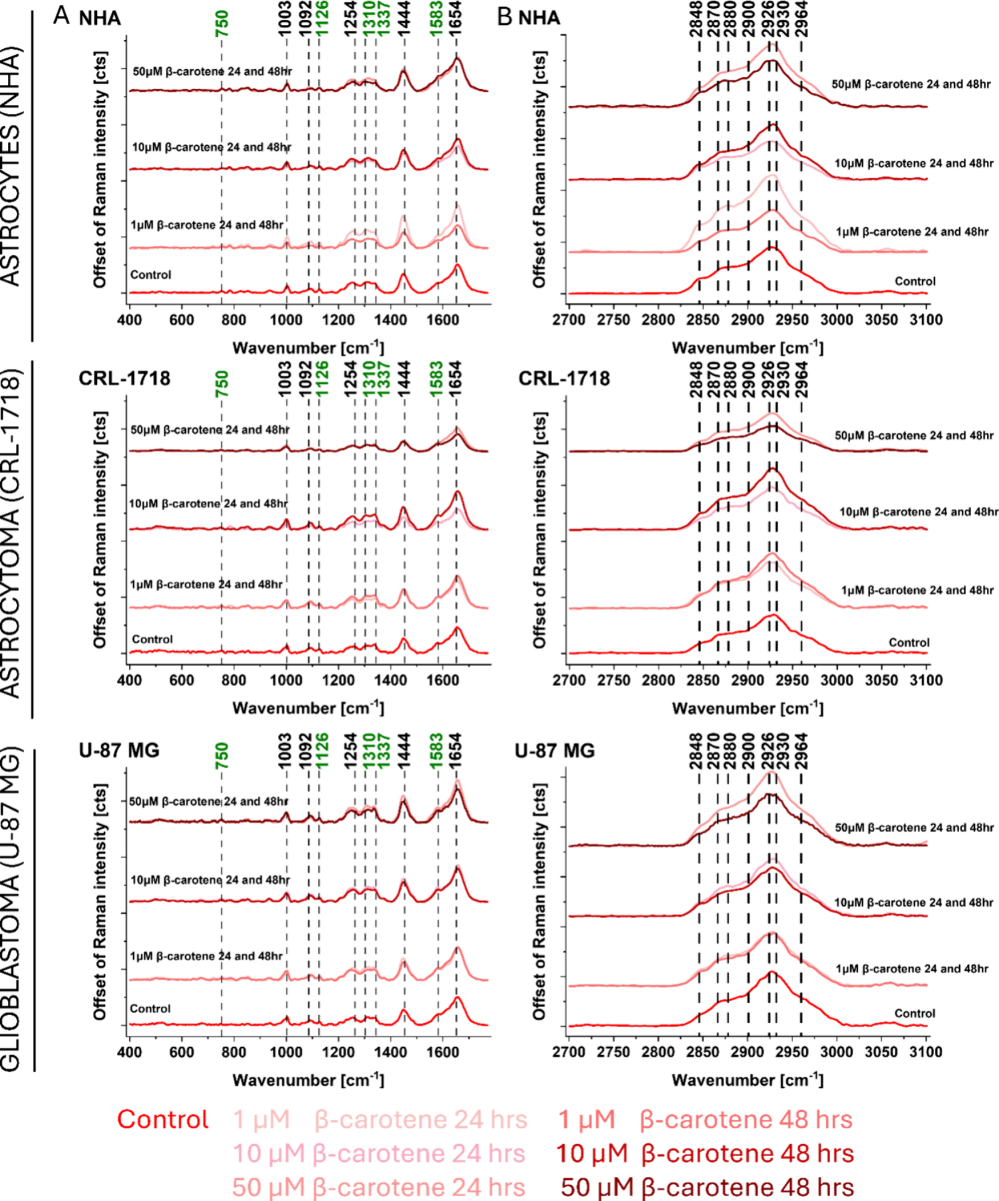
Offset of an average Raman spectrum for cell nucleus (NHA, CRL-1718, and U-87 MG cells). Ranges: (A) 400–1800 cm^–1^ and (B) 2700–3100 cm^–1^. Colors on the spectra correspond to the control and supplemented cells: 1, 10, and 50 μM of β-carotene after 24 and 48 h. Cytochrome *c* is represented by the Raman bands marked in green.

**Figure 6:**
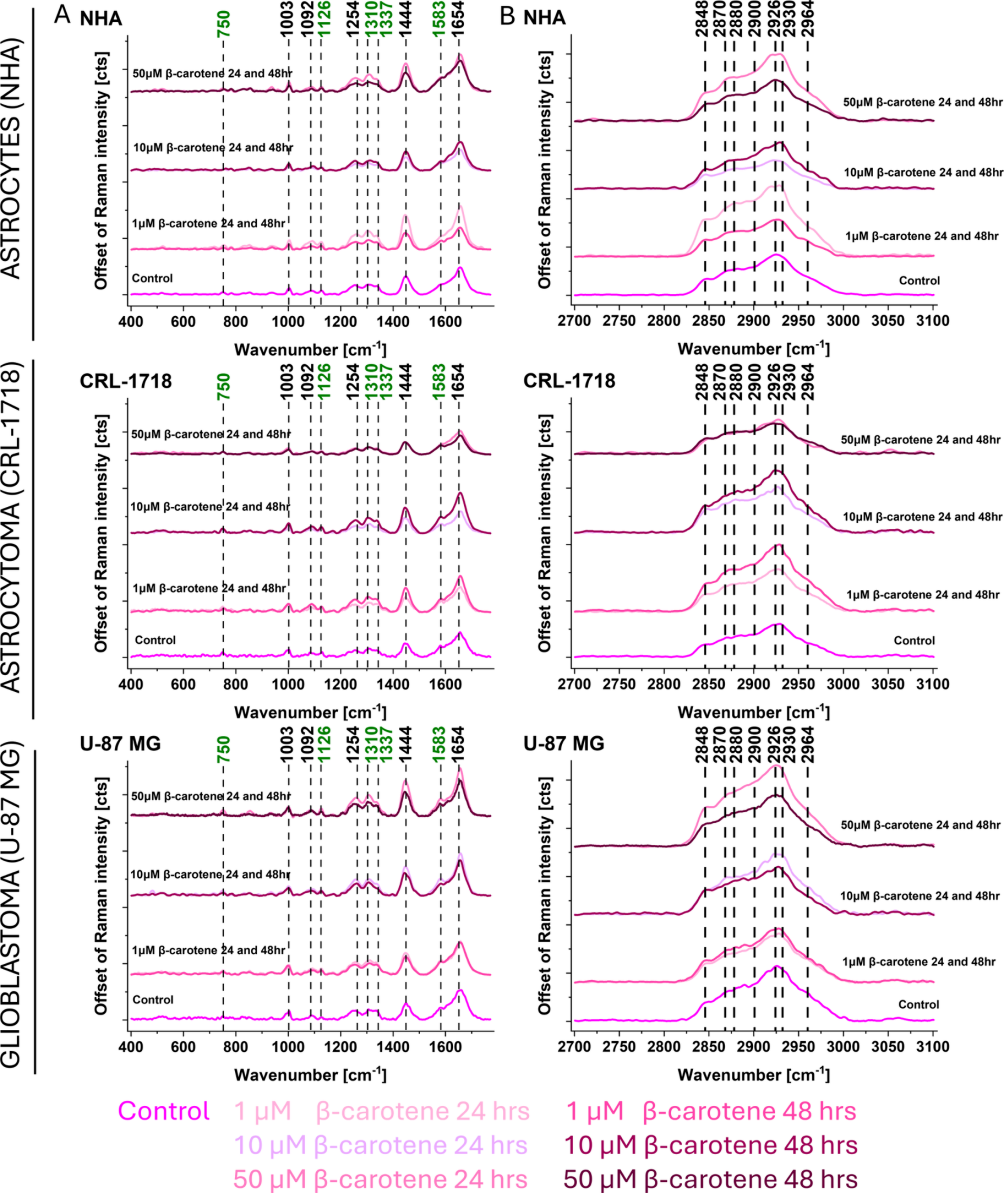
Offset of an average Raman spectrum for cell mitochondria (NHA, CRL-1718, and U-87 MG cells). Ranges: (A) 400–1800 cm^–1^ and (B) 2700–3100 cm^–1^. Colors on the spectra correspond to control and supplemented cells: 1, 10, and 50 μM of β-carotene after 24 and 48 h. Cytochrome *c* is represented by the Raman bands marked in green.

Figure 4 shows that the intensity of Raman bands of lipid droplets/endoplasmic reticulum changed after supplementation with β-carotene. Raman spectra of normal human astrocytes showed higher intensity of all bands after 24 h of incubation with β-carotene apart from 10 μM of β-carotene. This is important because it may indicate that lipid production increased after the addition of β-carotene, which may be related to changes in cell metabolism. In cells of the CRL-1718 line, the most significant increase in band intensity can be observed after supplementation with 1 μM of β-carotene after 48 h. For the U-87 MG cell line with 10 and 50 μM β-carotene, higher-intensity Raman signals are observed for 24 h of incubation and 1 μM after 48 h of incubation. The main differences are present for Raman bands at: 1310 (DNA/cytochrome *c*), 1337 (lipids and proteins), 1444 (lipids), 1583 (cytochrome *c*), 1654 (lipids and proteins), 2848 (lipids), and 2930 (lipids and proteins) cm^–1^. The intensities of bands at 750, 1126, and 1254 cm^–1^ have not changed significantly when comparing incubation times. Bands at 2848 and 2930 cm^–1^ correspond to CH_2_ stretch in lipids/fatty acids, CH_2_ stretch in phospholipids and proteins, and CH_3_ stretch in proteins.^62^ This provides evidence that the most significant changes after supplementation have occurred in bands assigned to lipids in lipid droplets, because they represent the primary lipid storage structures within the cell and serve as a sensitive indicator of lipid metabolism and dynamics at the subcellular level. It is worth noting that cancerous cells expressed a higher intensity of lipid droplet bands after 48 h with 50 μM of β-carotene than normal human astrocytes. Figures 5 and 6 and Figure S1 present the offset of Raman intensity in cell nucleus (Figure 5), mitochondria (Figure 6), and cytoplasm (Figure S1) for NHA, CRL-1718 and U-87 MG cell lines. The changes of Raman bands intensity observed in the cell nucleus, mitochondria, and cytoplasm were comparable to changes in lipid droplets/endoplasmic reticulum presented in Figure 4. Observed changes in the cytoplasm were less noticeable than in lipid droplets, cell nucleus, and mitochondria. To verify why we did not observe Raman bands of β-carotene at 1005, 1156, and 1523 cm^–1^ (Figure 1) in the supplemented cells’ spectra, we prepared a calibration curve determining the minimum concentration of Raman detection. Figure 7 shows a calibration curve for β-carotene solutions from 1 to 10 mM of the strongest Raman band at 1523 cm^–1^.

**Figure 7:**
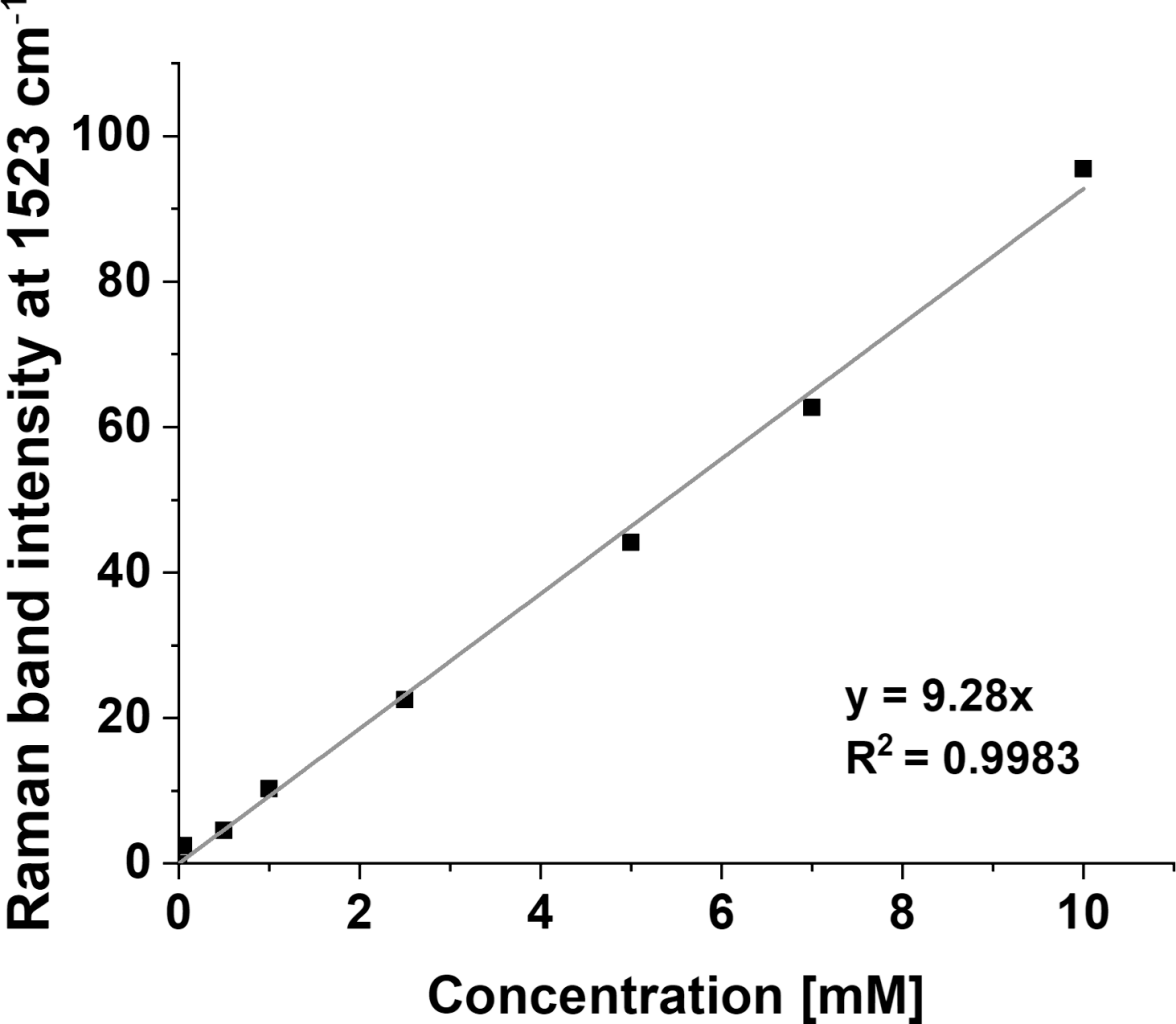
Determination of the minimum detectable concentration of β-carotene using RS based on the band intensity at 1523 cm^–1^. The β-carotene stock solution was prepared at a concentration of 10 mM in DMSO and subsequently diluted to the desired concentrations using a serial dilution method.

One can see from Figure 7 that the limitation of RS in the detection of β-carotene is an ∼0.5 mM concentration. There are reports in the literature that β-carotene can be detected by spectroscopic methods at lower concentrations.^63,64^ However, under our measurement conditions, on the equipment we used, the minimum concentration is about 0.5 mM. Now let us show difference spectra to explain the changes observed in Figures 4–6 and Figure S1. The obtained results for fingerprint and high-frequency regions are presented in Figure 8.

**Figure 8:**
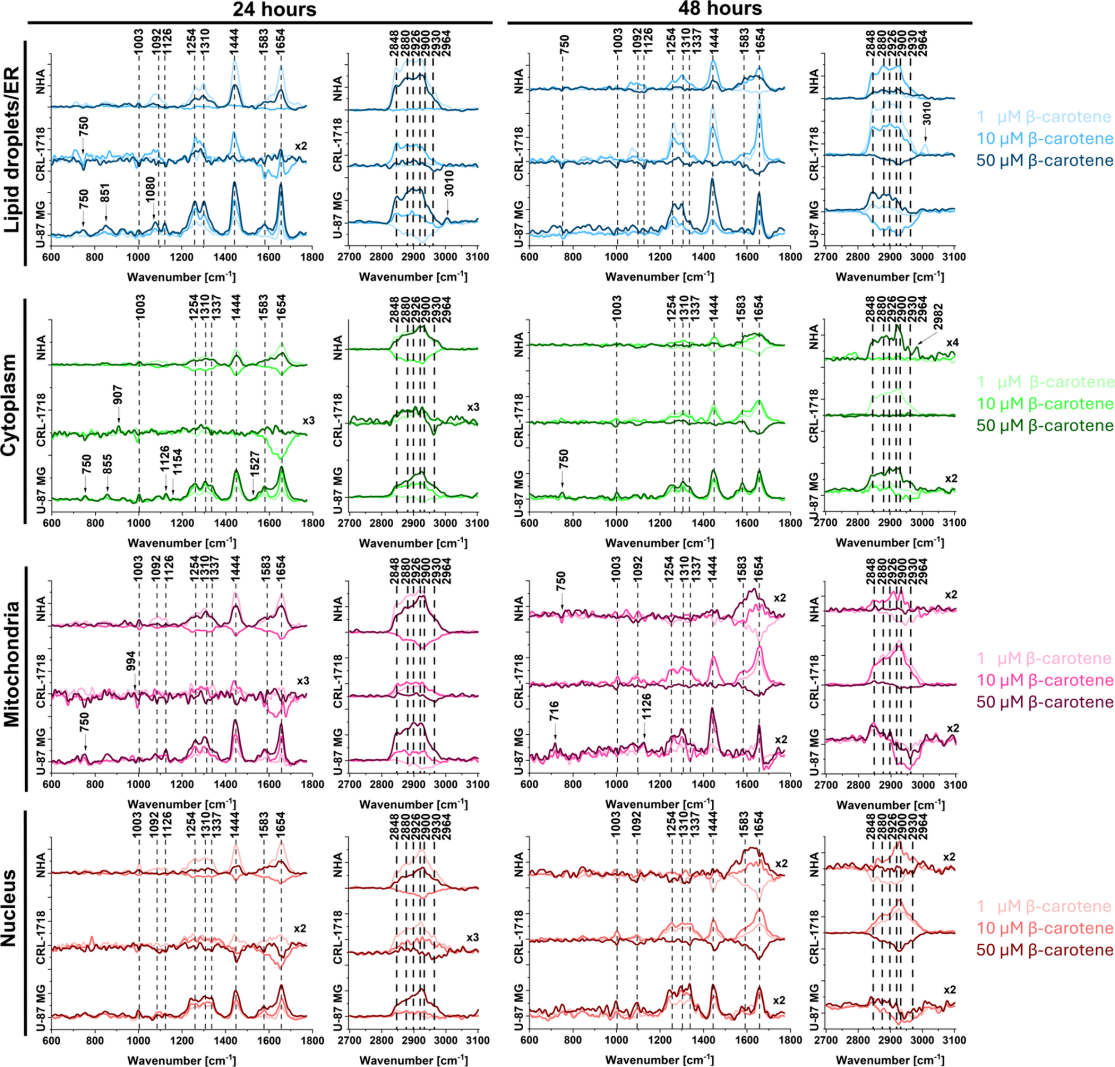
Offset of difference Raman spectra for cell organelles in NHA, CRL-1718, and U-87 MG cells in a range from 400 to 1800 cm^–1^ and 2700 to 3100 cm^–1^. Colors on the spectra correspond to lipid droplets/endoplasmic reticulum (blue), mitochondria (magenta), cytoplasm (green), and nucleus (red) for each variant of supplementation: 1, 10, and 50 μM of β-carotene after 24 and 48 h.

Figure 8 presents an offset of difference Raman spectra in the range from 400 to 1800 cm^–1^ and from 2700 to 3100 cm^–1^. To explain the effect of β-carotene supplementation, presented data was obtained as a result of the subtraction of control cells from supplemented cells at the concentrations of 1, 10, and 50 μM after 24 and 48 h. The intensity of all Raman bands for each cell line changes between 24 and 48 h of incubation with β-carotene.

The intensity of the band at 750 cm^–1^ was lower than the control in lipid droplets after 48 h of supplementation in CRL-1718 and U-87 MG cell lines. The band at 1003 cm^–1^ changed in the nucleus (24 and 48 h), lipid droplets (24 and 48 h), and mitochondria after 48 h. In lipid droplets, we observed lower intensity (CRL-1718 and U-87 MG) and higher in mitochondria and nucleus (all cell lines). The intensity of the band at 1092 cm^–1^ was higher in all organelles, except the cytoplasm for all cell lines. The band at 1126 cm^–1^ had higher intensity in lipid droplets (24 and 48 h), mitochondria, and nucleus (24 h) for NHA and U-87 MG cells and lower for CRL-1718 cells. The intensity of bands at 1254 and 1310 cm ^–1^ were higher for all organelles of NHA and U-87 MG cells after 24 h. For CRL-1718 cells after 24 h, the increase was negligible. After 48 h in NHA cells only in lipid droplets, the intensity of bands at 1254 and 1310 cm^–1^ was higher, and in mitochondria, cytoplasm, and nucleus, changes were minor. The band at 1337 cm^–1^ had higher intensity in mitochondria, cytoplasm, and nucleus of all cell lines. In CRL-1718, the changes were less noticeable. The intensity of the band at 1444 cm^–1^ was higher for all organelles and cell lines except CRL-1718 after 24 h of incubation. The intensity of bands at 1583 and 1654 cm^–1^ was significantly higher in most organelles and cell lines except CRL-1718 after 24 h of supplementation, where the changes were minor, and 50 μM of β-carotene after 48 h, where the intensity was lower than in control cells. The intensity of the band at 2848 cm^–1^ was higher than in control cells for most organelles apart from NHA mitochondria, nucleus, and cytoplasm after 48 h of incubation. The band at 2880 cm^–1^ had a higher intensity in most of the organelles and cell lines except the cytoplasm, where changes were negligible. The intensity of bands at 2900, 2926, and 2930 cm^–1^ was higher in most organelles and cell lines except lower intensity after 48 h in mitochondria, cytoplasm, and nucleus of the U-87 MG cell line. What is important, 50 μM of β-carotene after 48 h of incubation resulted in a slight change in lipid droplets of NHA cells, a minor lower intensity in CRL-1718 cells and significantly higher intensity in U-87 MG cells. It should be pointed out that the difference spectra reveal that new Raman bands at: 716 (lipids and proteins), 851/855 (polysaccharides or proteins), 907 (formalin contamination), 994 (sugar ribose), 1080 (phospholipids), 1154 (β-carotene), 1527 (β-carotene), 2964 (CH_3_ stretch band), and 3010 cm^–1^ (unsaturated =CH stretch) were observed. Within the identified Raman bands, the ones at 1154 and 1527 cm^–1^ can be directly linked to β-carotene.

There was a significant correlation between the cell line and the response to supplementation. Thus, it might indicate changes in lipids, proteins, and cytochrome *c*. Deeper insight into presented changes showed that there is a correlation between observed changes and cell organelle, which shows that β-carotene supplementation alters metabolism in lipid droplets/endoplasmic reticulum and mitochondria. β-Carotene possesses antioxidant properties. While not directly related to lipid synthesis, these properties could influence cellular signaling pathways that indirectly impact lipid metabolism.

Concerning the outcomes presented so far, it can be suggested that depending on whether the cell is normal or cancerous, the effect of β-carotene supplementation is different. In each of the cell organelles presented in Figures 4–6 and 8 and Figure S1, changes in the Raman lipid band vibration at 1444 cm^–1^ were noticeable, thereby one-way ANOVA analysis of the intensity of this band was performed (Figure 9).

**Figure 9:**
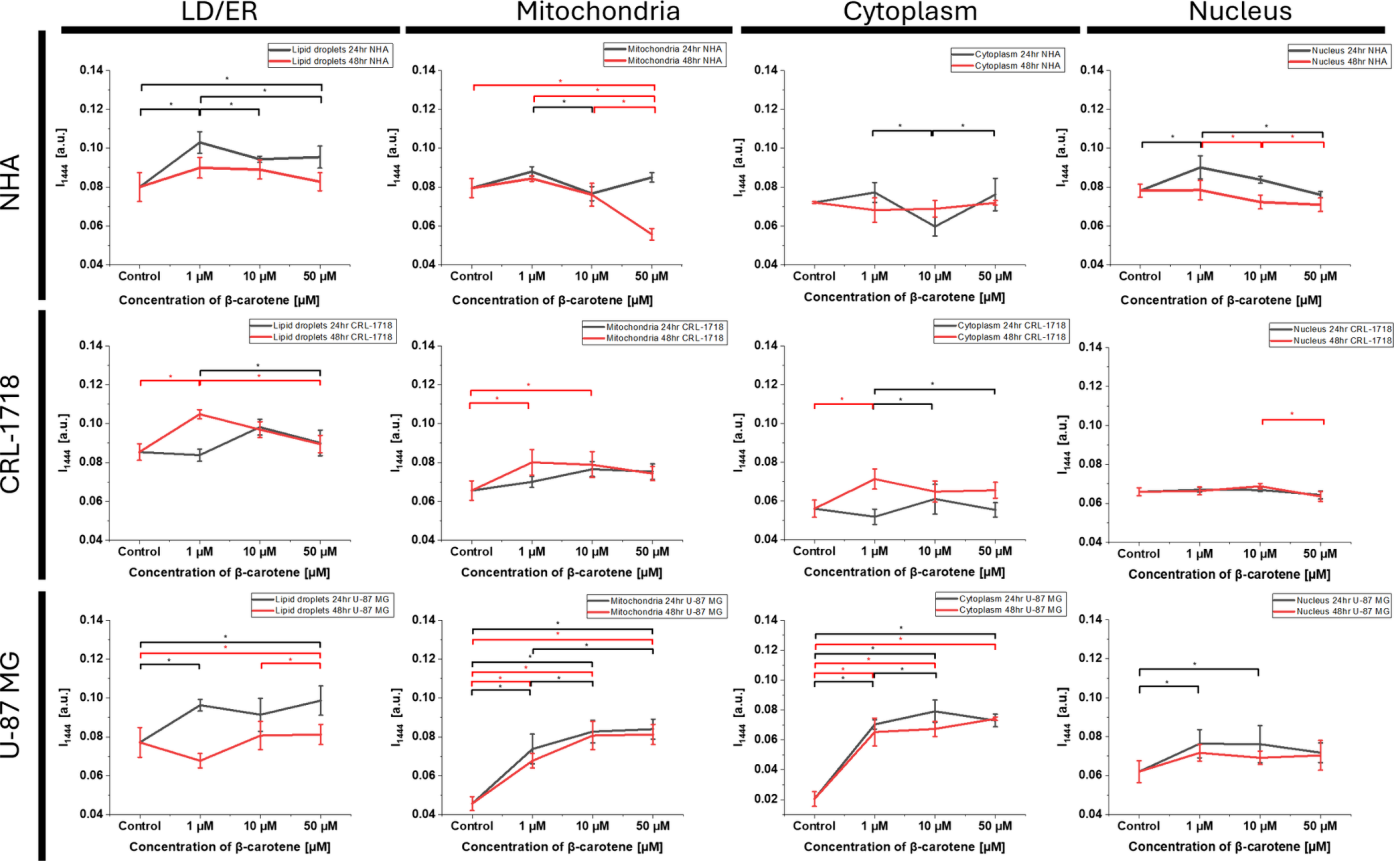
Differences in the Raman band at 1444 cm^–1^ analyzed by one-way ANOVA. Raman peak intensity and standard deviation (SD) for lipid droplets/endoplasmic reticulum, mitochondria, cytoplasm, and nucleus in NHA, CRL-1718, and U-87 MG cell lines. Black color refers to average data for cells supplemented for 24 h and red color for cells supplemented for 48 h. Points represent control (unsupplemented cells) and cells supplemented with 1, 10, and 50 μM of β-carotene. Significant differences are presented with asterisk *.

Figure 9 presents the ANOVA analysis of the Raman band at 1444 cm^–1^ for lipid droplets, mitochondria, cytoplasm, and nucleus in NHA, CRL-1718, and U-87 MG cell lines. The band at 1444 cm^–1^ corresponds to lipids, CH_2_ scissoring deformation (δCH_2_).^65^ Raman spectra were normalized by a vector norm in Origin Pro. The Raman intensity of the band at 1444 cm^–1^ of the NHA cell line was higher after supplementation for 24 h than after 48 h in all analyzed organelles. In lipid droplets and the nucleus of NHA cells, supplementation caused a higher intensity of the band at 1444 cm^–1^. In mitochondria, supplementation with 10 and 50 μM β-carotene resulted in a lower intensity than for control cells. Changes in the intensity of the band at 1444 cm^–1^ in the cytoplasm were minor. The β-carotene supplementation of CRL-1718 cells resulted in a higher intensity of the band at 1444 cm^–1^ for 1 μM and slightly higher for 10 and 50 μM in lipid droplets, mitochondria, and cytoplasm. Changes in the CRL-1718 cell’s nucleus were minor. The most significant differences can be observed in the U-87 MG cell line. The intensity of the Raman band at 1444 cm^–1^ was much lower in the control (unsupplemented) than in the supplemented cells. Significant differences are presented in mitochondria and the cytoplasm, which might be the result of the redirection of the metabolism to de novo lipid production. De novo lipid synthesis reduces the reliance on oxidative phosphorylation, possibly inhibiting this pathway and promoting glycolysis through the Warburg effect, which might enhance cancer development. Further investigation is required to clarify the role of lipid production in brain cancer cells. The U-87 MG is the most aggressive cell line. Thus, the impact of β-carotene might be related to the aggressiveness of cancer cells. It should be mentioned that the differences observed in mitochondria and cytoplasm might result from the changes in the amount of reactive oxygen species (ROS) due to β-carotene antioxidant activity.^66–68^ Interestingly, we did not observe notable changes in the nucleus.

## Discussion

The presented results so far expand the current state of knowledge on the impact of β-carotene on the metabolism of brain cancer cells. Changes in intensity of particular Raman bands (1444, 1654 cm^–1^) provide evidence that β-carotene influences cell metabolism, especially by increasing the amount of lipids in the most aggressive cancer cells (U-87 MG) and this alteration can be observed using Raman imaging and spectroscopy. It might be connected to de novo lipid synthesis.

Summarizing our observations indicated that after supplementation, the Raman band intensities were greater, with the increase being particularly noticeable in certain bands as the β-carotene concentration increased: 1310, 1337, 1444, 1583, and 1654 cm^–1^, which correspond to cytochrome *c* (1310 and 1583 cm^–1^), lipids (1444 cm^–1^), and lipids/proteins (1337 and 1654 cm^–1^). The Raman bands identified in this study are prominently represented within the latent variables derived from PLS-DA (Figure S4). These spectral features effectively discriminate between normal and cancerous brain cells under both experimental conditions: nonsupplemented (control) and β-carotene-supplemented. PLS-DA results for control cells and those treated with 50 μM β-carotene for 24 and 48 h are presented in Figure S4, showing a clear separation between control and supplemented cells, as well as between the two incubation times. Table S1 summarizes the Pearson correlation coefficients between control samples (each organelle within each cell line) and cells exposed to β-carotene supplementation (1, 10, and 50 μM) for 24 and 48 h. Across all three cell lines (NHA, CRL-1718, and U-87 MG), the highest correlation coefficients were consistently observed among the nucleus, mitochondria, cytoplasm, and cell membrane at lower supplementation levels (1 and 10 μM). Pronounced deviations emerged primarily at the highest concentration (50 μM), particularly within the lipid droplet/endoplasmic reticulum (LD/ER) region, where correlation values markedly decreased. Lower Pearson correlation coefficients indicate more pronounced biochemical differences resulting from the supplementation. Notably, U-87 MG cells exhibited the greatest variability with reduced correlations in the mitochondria and LD/ER at 50 μM, suggesting a more heterogeneous biochemical response to β-carotene. In contrast, NHA and CRL-1718 cells maintained relatively stable correlation profiles across most organelles and supplementation conditions. This suggests that β-carotene supplementation modulates the overall cellular metabolism by impacting the abundance and structural organization of critical biomolecules, including lipids and proteins. Such alterations may influence membrane dynamics, signaling pathways, and mitochondrial function, thereby affecting key cellular processes like energy production, oxidative stress response, and apoptosis. Consequently, these biochemical changes reflected in the Raman spectra underscore the multifaceted role of β-carotene in regulating tumor cell physiology. Conducted experiments on mice by Nishino et al.^69^ have proved that consumption of carotenoids prevents from cancer but did not provide any explanation for the mechanisms that they observed. Kim et al. have demonstrated that β-carotene inhibits neuroblastoma cell invasion and metastasis in vitro and in vivo by decreasing the level of hypoxia-inducible factor-1α.^70^ As presented in our paper, alterations of metabolism in cancer cells might be related to Nishino and Kim’s observations. It has been proven by Malvy et al.^71^ that childhood cancer patients have lower blood levels of β-carotene. Aggarwal et al.^72^ demonstrated that β-carotene levels are reduced in brain tumor tissues compared to healthy controls. This observation is consistent with our findings, which indicate that normal human astrocytes (NHA) metabolize β-carotene differently from cancer cell lines (CRL-1718 and U-87 MG). Notably, the more aggressive the cancer phenotype, the more rapidly β-carotene appears to be converted to downstream metabolites. These results support the notion that carotenoid metabolism varies between normal and malignant cells, leading to measurable differences in intracellular β-carotene levels. Furthermore, β-carotene has been found as an important factor in neurodegenerative diseases.^24^ Moreover, carotenoids tend to be important in elderly brain function.^73^ These two cases might be connected to changes in metabolism shown in our work and the result that β-carotene is converted to other compounds, which build brain structures or improve brain functioning. Additional study is essential to gain a better understanding of the relationship between β-carotene and brain function, and our research provides valuable groundwork for future consideration.

## Conclusions

In this paper, we presented application of Raman microspectroscopy imaging for monitoring metabolic changes in single normal and cancerous brain cells after β-carotene supplementation. Based on the Raman signatures observed, we determined that major alterations occurred in the lipid droplets/endoplasmic reticulum and mitochondria. Summarizing our findings, it was observed that following supplementation, the Raman band intensities increased, notably at 1310, 1337, 1444, 1583, and 1654 cm^–^^1^. These bands are linked to cytochrome *c* (1310, and 1583 cm^–^^1^), lipids (1444 cm^–^^1^), and lipids/proteins (1337 and 1654 cm^–^^1^). Thus, it can be concluded that the significant changes postsupplementation occurred in lipids and proteins. The most significant correlation between vibrational features and the response to supplementation was found for the U-87 MG line, which is the most aggressive cell line. Significant differences are presented in mitochondria and cytoplasm, which might be the result of redirection of the metabolism to de novo lipid production. Thus, the impact of β-carotene might be related to the aggressiveness of cancer cells. It should be mentioned that the differences observed in mitochondria and cytoplasm might result from changes in the amount of reactive oxygen species (ROS) due to β-carotene antioxidant activity.

Overall, more research is needed to understand the specific effects of carotene on the lipid droplet synthesis. The current understanding suggests an indirect influence through vitamin A conversion and potential modulation of cellular signaling, but the exact mechanisms remain elusive. For the future, we suggest biological studies on the mechanism of action of β-carotene in human brain cancer cells.

## Supplementary Material



## Notes

Four additional figures and one table: Figure S1 presents offset average Raman spectra of the cytoplasm for NHA, CRL-1718, and U-87 MG cells; Figure S2 shows Raman images, Raman maps, and representative spectra of cells supplemented with β-carotene after 48 h of incubation; Figure S3 provides a quantitative comparison of cell area under control conditions and after β-carotene treatment at different concentrations, following 24 and 48 h incubation periods; Figure S4 shows PLS-DA results of control cells and cells supplemented with 50 μM β-carotene for 24 and 48 h; Table S1: Pearson correlation coefficients between average Raman spectra of individual organelles in control NHA, CRL-1718, and U-87 MG cells and the corresponding organelles in supplemented cells (PDF)
