# Supplementary material for: Raman Spectroscopy and Imaging Reveal the Effect of β‑Carotene Supplementation on Brain Cancer Cells
Source: Biochemistry. 2025 Aug 20;64(17):3745–59. doi: 10.1021/acs.biochem.5c00231 (PMC12409892; doi:10.1021/acs.biochem.5c00231)
Supplement: Supplementary file 1 [file bi5c00231_si_001.pdf]

Supporting Information

Raman spectroscopy and imaging reveal the effect of β-carotene supplementation on brain cancer cells

Karolina Jarczewska[a], Monika Kopeć[a], Halina Abramczyk[a], Jakub Maciej Surmacki[a], *

[a]Lodz University of Technology, Faculty of Chemistry, Institute of Applied Radiation Chemistry, Laboratory of Laser Molecular Spectroscopy, Wroblewskiego 15, 93-590 Lodz, Poland

* Correspondence: jakub.surmacki@p.lodz.pl; Tel.: +48426313188

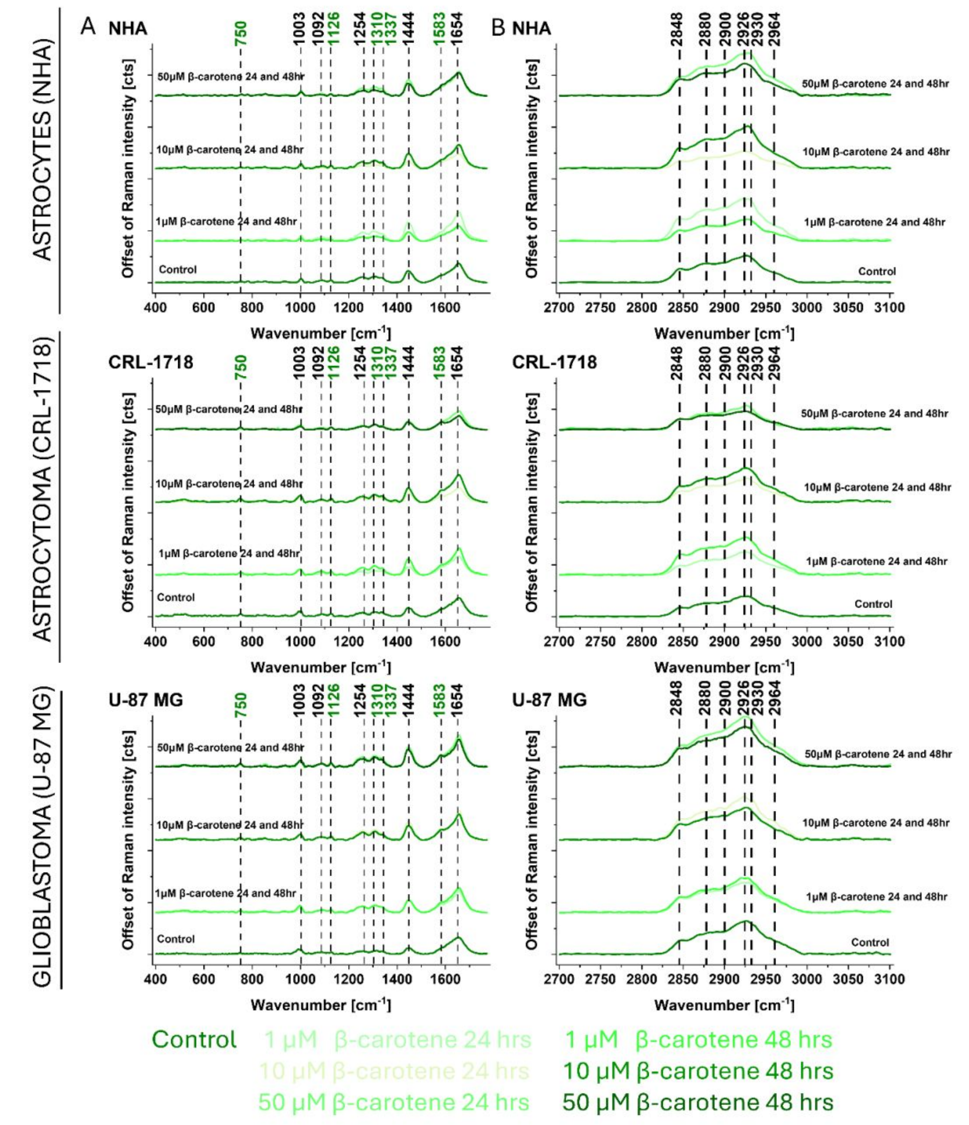

Figure S1. *The offset of an average Raman spectra for cell cytoplasm (NHA, CRL-1718 and U-87 MG cells). Ranges: (A) 400-1800 cm*$^{-1}$ *and (B) 2700-3100 cm*$^{-1}$*. Colors on the spectra correspond to control and supplemented cells: 1, 10 and 50 µM of* β*-carotene after 24 and 48 hours. Cytochrome c is represented by the Raman bands marked in green.*

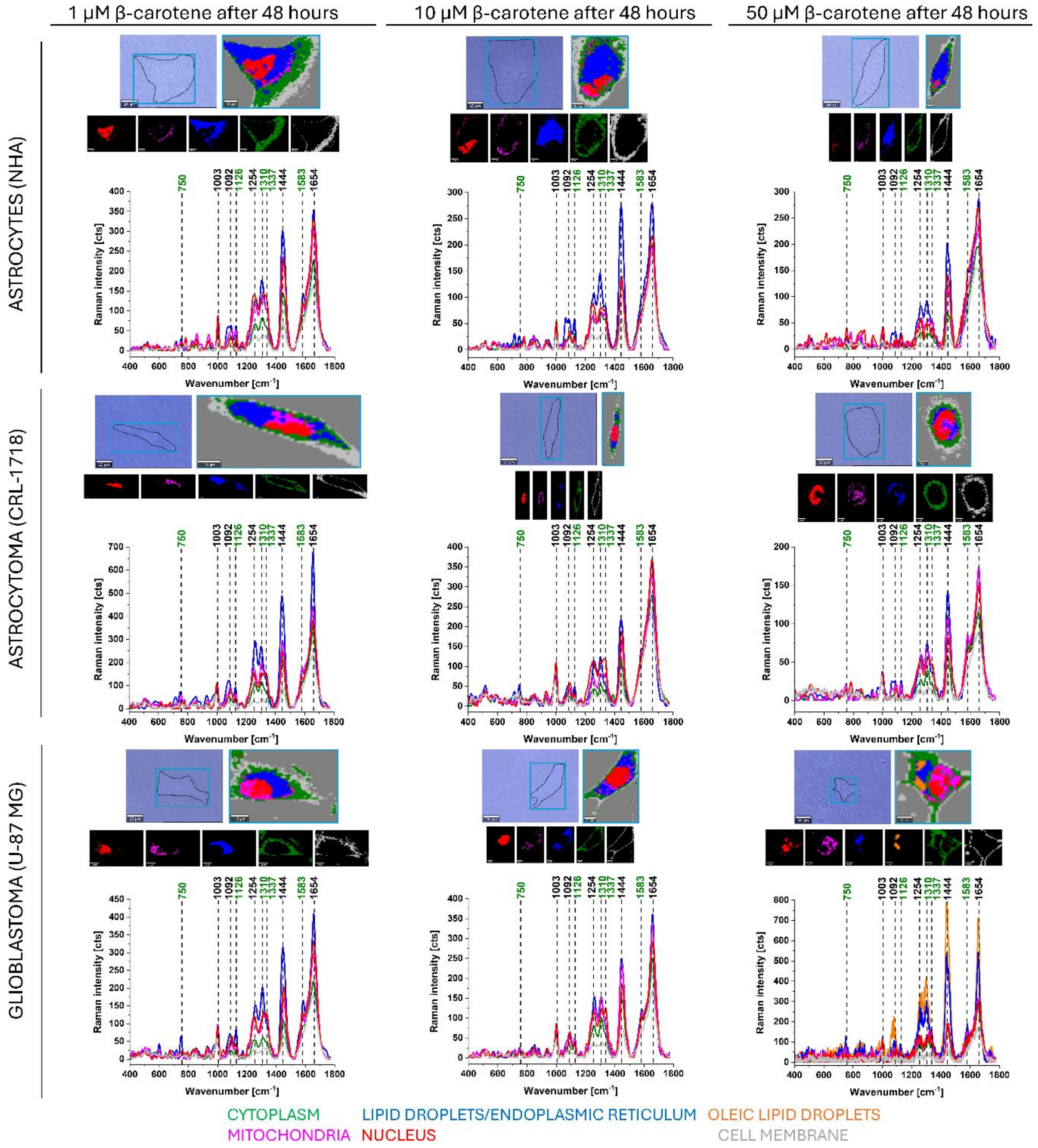

Figure S2. *Typical Raman imaging of a normal human astrocyte (NHA), astrocytoma (CRL-1718) and glioblastoma (U-87 MG) cells supplemented with* β*-carotene at concentrations: 1, 10, 50 µM and time of incubation 48 hours. Microscope images of the cell; Raman images of the cell; Raman images of particular cell organelles; representative Raman spectra of each cell organelle: nucleus (red), mitochondria (magenta), lipid droplets/endoplasmic reticulum (blue), oleic lipid droplets (orange), cytoplasm (green) and cell membrane (light grey). Raman spectra were measured in the range 400-1800 cm*$^{-1}$ *with an integration time of 0.5 seconds at 10 mW and 532 nm. Colors on the spectra correspond to colors on Raman images.*

Figure S3 displays a quantitative comparison of the cell area under control conditions and after treatment with various concentrations of β-carotene, following 24- and 48-hour incubation periods. A consistent trend toward reduced average cell size is observed in β-carotene-treated cells compared to the untreated controls, particularly after 48 hours and at higher concentrations. These observations suggest that β-carotene supplementation may influence cellular morphology, possibly reflecting changes in proliferation rate, cytoskeletal organization, or stress response.

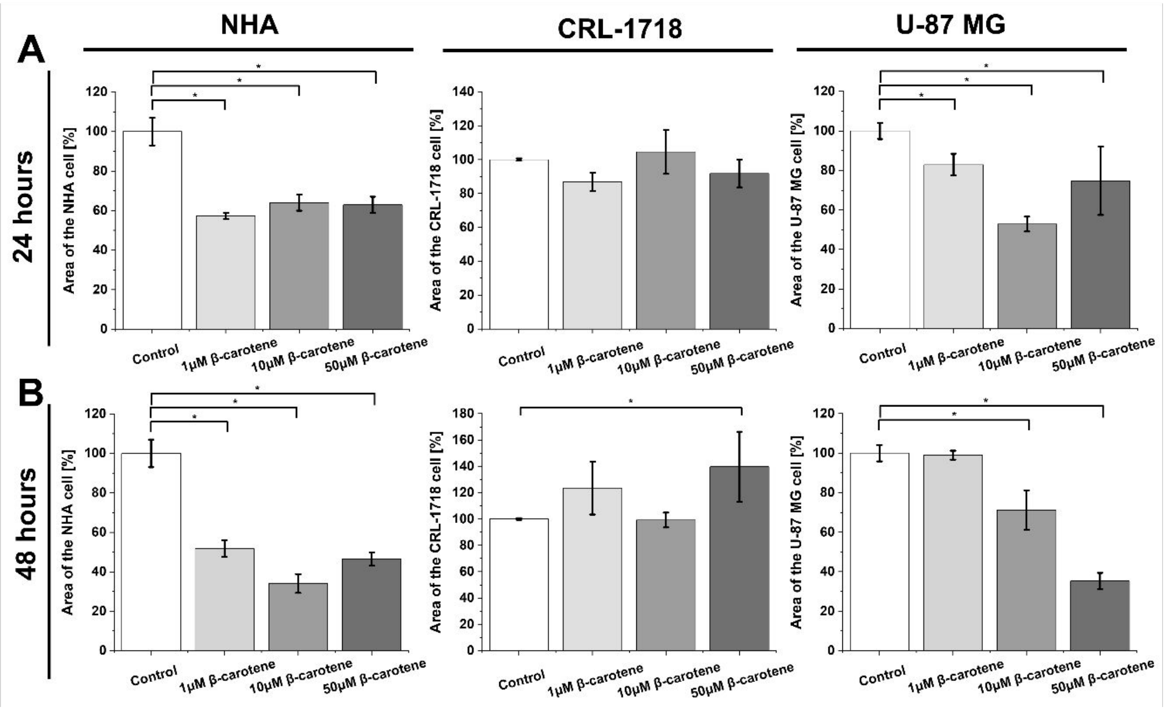

Figure S3. *Quantitative analysis of cellular area before supplementation (white) and following treatment with β-carotene at concentrations of 1 μM (light grey), 10 μM (grey), and 50 μM (dark grey). Statistically significant differences ($p \leq 0.05$) are indicated by an asterisk *.*

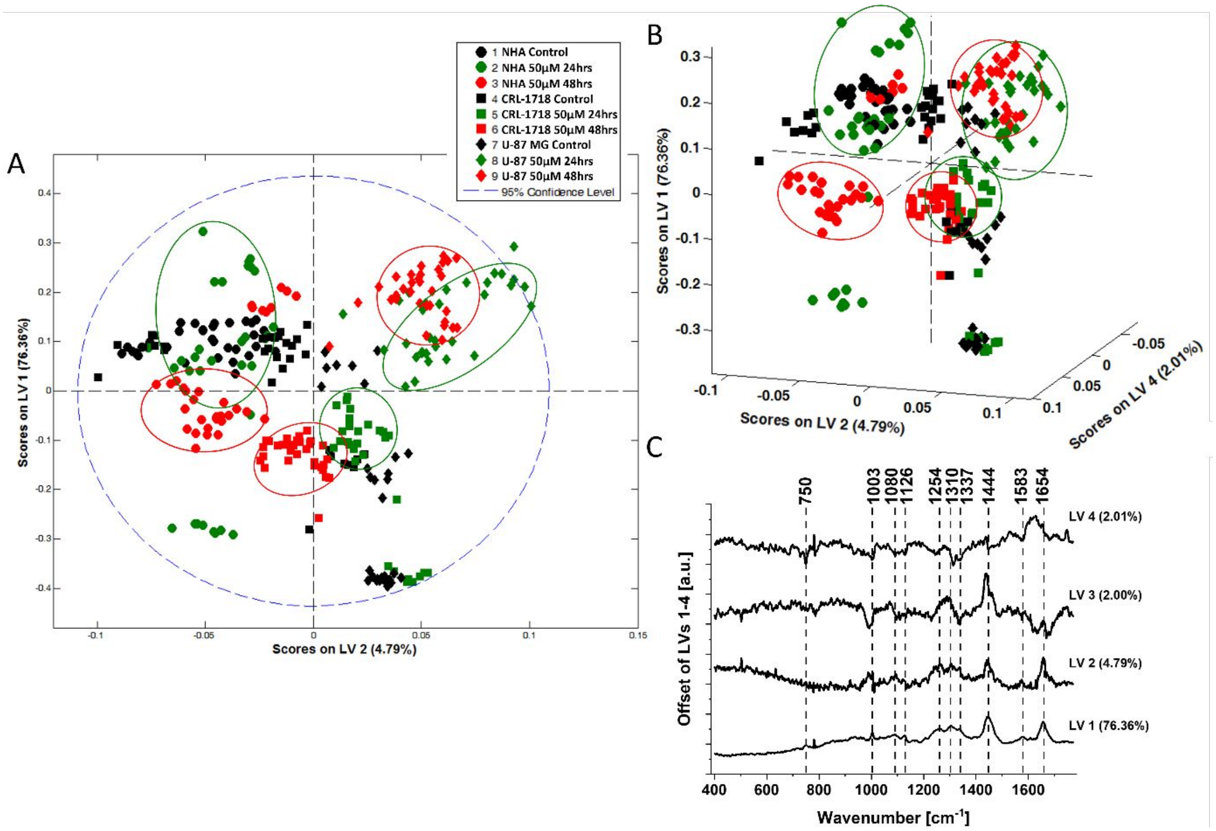

Figure S4. *PLS-DA score plots: (A) LV1 vs. LV2, (B) LV1 vs. LV2 vs. LV4 for Raman spectra of control cells (black): NHA (circle), CRL-1718 (square), U-87 MG (rhombus) and supplemented with β-carotene in concentration of 50 μM for 24 hours (green) and 48 hours (red): NHA (circle), CRL-1718 (square), U-87 MG (rhombus). (C) The plots of latent variables (LV1-4). Each point represents the average line Raman spectrum across a single cell. Spectra were vector normalized within the range of 400–1800 cm^{-1} and mean centered.*

Table S1. *Pearson correlation coefficients between average Raman spectra of individual organelles in control NHA, CRL-1718, and U-87 MG cells and the corresponding organelles in supplemented cells.*

		1 µM 24hrs	1 µM 48hrs	10 µM 24hrs	10 µM 48hrs	50 µM 24hrs	50 µM 48hrs
**NHA**	**Nucleus**	0.99280	0.99771	0.99783	0.99591	0.99723	0.97369
	**Mitochondria**	0.98534	0.99483	0.99636	0.99197	0.99290	0.97472
	**LD / ER**	0.97435	0.99666	0.99685	0.99126	0.98875	0.98877
	**Cytoplasm**	0.99536	0.99714	0.97936	0.99812	0.99760	0.98650
	**Cell membrane**	0.99739	0.99651	0.99612	0.99573	0.99566	0.98704
**CRL-1718**	**Nucleus**	0.98967	0.97877	0.97385	0.98157	0.98967	0.98758
	**Mitochondria**	0.98986	0.97158	0.97852	0.98550	0.98952	0.98624
	**LD / ER**	0.99065	0.91087	0.93604	0.97094	0.98826	0.97672
	**Cytoplasm**	0.99556	0.97667	0.98188	0.99377	0.99424	0.98286
	**Cell membrane**	0.99162	0.99210	0.97940	0.99335	0.99144	0.98904
**U-87 MG**	**Nucleus**	0.96749	0.99299	0.96779	0.97534	0.97016	0.97341
	**Mitochondria**	0.96656	0.99189	0.95580	0.96471	0.93917	0.95914
	**LD / ER**	0.95998	0.99272	0.96070	0.97861	0.94702	0.92781
	**Cytoplasm**	0.95042	0.98287	0.91962	0.95608	0.93641	0.93676
	**Cell membrane**	0.97309	0.98209	0.95206	0.96178	0.96564	0.96498
